# A Trajectory and Orientation Reconstruction Method for Moving Objects Based on a Moving Monocular Camera

**DOI:** 10.3390/s150305666

**Published:** 2015-03-09

**Authors:** Jian Zhou, Yang Shang, Xiaohu Zhang, Wenxian Yu

**Affiliations:** 1 Shanghai Key Laboratory of Navigation and Location Based Services, Shanghai Jiao Tong University, Shanghai 200240, China; E-Mail: zhou_jian_1980@foxmail.com; 2 College of Aerospace Science and Engineering, National University of Defense Technology, Changsha 410073, China; E-Mails: shangyang1977@nudt.edu.cn (Y.S.); zhangxiaohu@nudt.edu.cn (X.Z.); 3 Shanghai Key Laboratory of Intelligent Sensing and Recognition, Shanghai Jiao Tong University, Shanghai 200240, China

**Keywords:** videometrics, monocular moving camera, moving object, necessary and sufficient condition

## Abstract

We propose a monocular trajectory intersection method to solve the problem that a monocular moving camera cannot be used for three-dimensional reconstruction of a moving object point. The necessary and sufficient condition of when this method has the unique solution is provided. An extended application of the method is to not only achieve the reconstruction of the 3D trajectory, but also to capture the orientation of the moving object, which would not be obtained by PnP problem methods due to lack of features. It is a breakthrough improvement that develops the intersection measurement from the traditional “point intersection” to “trajectory intersection” in videometrics. The trajectory of the object point can be obtained by using only linear equations without any initial value or iteration; the orientation of the object with poor conditions can also be calculated. The required condition for the existence of definite solution of this method is derived from equivalence relations of the orders of the moving trajectory equations of the object, which specifies the applicable conditions of the method. Simulation and experimental results show that it not only applies to objects moving along a straight line, or a conic and another simple trajectory, but also provides good result for more complicated trajectories, making it widely applicable.

## 1. Introduction

Most reconstruction methods for three-dimensional objects by a monocular moving camera require a static scene where no objects move within the sequence of images taken by the camera. Based on the assumption the scene remains static, even when the camera parameters are unknown, the results of scene reconstruction with only a scale factor difference from the actual three-dimensional structures can also be determined by the multi-view geometric constraint. In recent years, mathematical theories related to three-dimensional reconstruction problems on static scenes have become well developed, allowing major advances in a variety of related applied research topics [1,2,3,4] that address a large number of demands such as virtual reality, the creation of digital artifacts, three-dimensional electronic maps of real scenes, and so on. These three-dimensional reconstruction methods on static scenes assist researchers in solving problems related to camera calibration on unknown scenes and for dense points matching. However, three-dimensional reconstruction work also uses the traditional camera measurement methods; that is, the camera captures images at different locations or at different times, and then the homologous points in two or more images are matched to form a single point intersection image which allows the reconstruction of a three-dimensional data of the scene.

However, during the actual measurement task, there is a problem in calculating both the motion data while at the same time working with three-dimensional reconstruction of moving objects when using a monocular moving camera. For instance, for robot tournaments, robot soccer and other applications, robots need to measure the trajectory, velocity, acceleration, position, orientation and other parameters of the soccer ball or the opposing robots. When no other measuring device is used, the monocular camera can only determine the connecting line between the camera’s optical center and the moving object on each moment and position, and that line is the line of sight. Obviously, because of the lack of constraints the traditional “point intersection” method cannot be used for three-dimensional reconstruction of moving objects when using a monocular camera. It is a very difficult problem that how one can estimate the three-dimensional position and orientation of moving objects when using a monocular sequence of images in the field of machine vision, artificial intelligence, and so on. This problem is more challenging than traditional three-dimensional reconstruction. These two problems are essentially different [5]. Ultimately, the difficulty is lack of constraints. Naturally, to increase constraints is the key to solve it. Without other measuring equipment, two types of additional constraints come to mind:

One way is to consider the shape of the moving trajectory as additional constraints. Avidan and Shashua proposed a “trajectory triangulation” method [6] for straight line [7] and conic [8], and also their necessary conditions:

(1) When the point moves along a straight line, at least five images at different times and positions are required.
(2) A conic trajectory needs at least nine similar images and an initial plane of motion. Furthermore, there is no definite solution when the trajectory of the optical center of the camera and the trajectory of the object are at the same quadric.

The other way is to consider the kinematics laws as adding additional constraints. Filtering methods such as Extended Kalman Filtering are used to calculate the results. Han and Kanade [9] studied the three-dimensional reconstruction of the point with linear motion and proposed a filtering method for solving camera parameters, static scene reconstruction and a moving point, simultaneously. Xu *et al.* [10] used a filtering method to estimate an initial value, which is then recalculated by using the Hidden Markov algorithm. Many similar approaches are discussed by others.

In addition, other methods can restrain not only the shape, but the kinematics laws of the object, such as research on the orbits of satellites in a gravitational field [11]. However, the adaptability of the method is too bad to fit general situations. In a word, there are still some difficulties to overcome in the methods mentioned above.

Firstly, the acquisition of an initial value is not easy, and an initial value without enough precision will lead to failure of any subsequent iteration or filtering. In general, these methods can only get good results with quite good initial values.

Secondly, those are theoretically possible to obtain, but hard in practice. The trajectories of the objects studied in those methods are too simple to work in practice. Natural motions tend to be much more complex than linear or conic motion in a plane, and existing methods fail to calculate the more complex ones.

Thirdly, most current research only considers objects as a single point and lose sight of their orientation, but orientation cannot be ignored in many cases. For example, The “PnP” problems of a rigid body, or a multi-view geometric application. The former need more than three known feature points on the body, and the latter requires much more unknown but homonymous points in different images such as the “eight-point algorithm” of Harley [12].These do not work in the cases which dissatisfy the conditions of “PnP” methods or “eight points algorithms”. For instance, so far no perfect solution can recover the three-dimensional motion as well as orientation when an object is only with less than three valid feature points regardless of known or not. The main contributions of this paper are:

(1) To expand the traditional “point intersection” to “trajectory intersection” for object points;
(2) To deduce the conditions of a unique “trajectory intersection” solution;
(3) To extend the method to get the orientations of a rigid body with poor *a priori* knowledge.

The proposed method considers the exponential term of time as a vector base which unifies the motion of object and the motion of camera as a time polynomial with finite orders. By doing this, the position, speed, acceleration and other motion parameters are the coefficients of the polynomial and can be solved by linear equations without any initial value or iteration. Also, complete mathematical proofs for the existence and uniqueness of the solution are given by analyzing the relationship of the polynomial order between object and camera. Furthermore, the orientations of a rigid body with poor *a priori* knowledge are acquired by using the method for several points, simultaneously.

Since camera parameters could be known under some circumstances such as UAVs which can give their position by GPS and orientation by gyroscope, and the segmentation of moving objects in a static scene is quite advanced, these two problems are considered resolved, so this paper will not discuss them. For the preliminary work of this paper, please refer to [13].

## 2. Problem Description and Methods

### 2.1. Problem Description

First of all, an assumption is made that the motion of an object can be defined by certain mathematical equations with unknown parameters. A monocular camera which motion is arbitrary but known tracks the object and takes a series of pictures individually and independently. The problem is to reconstruct the three-dimensional trajectory, motion parameters, and orientation of the object based on the known camera and image sequence.

### 2.2. Point Models

**Figure 1:**
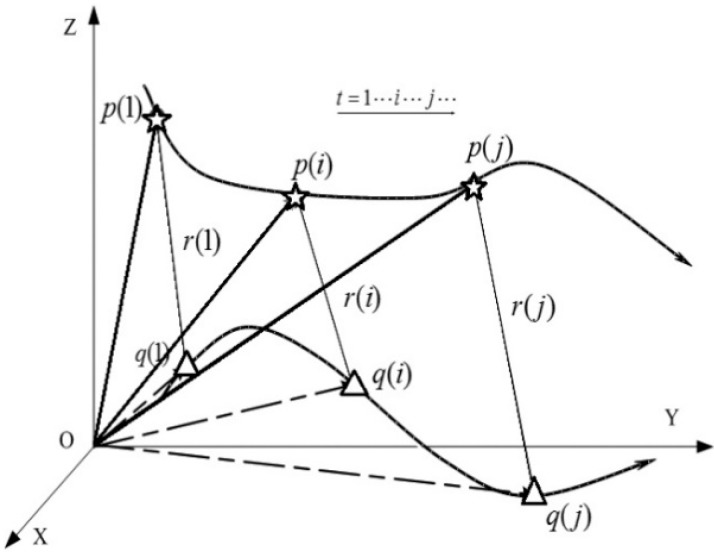
Point reconstruction principle.

At first, the object is considered as a point (Figure 1). The three-dimensional vector functions *q*(*t*) and *p*(*t*) in the Cartesian coordinate system represent the trajectory of the moving point and the optical center of the camera within a period of time, respectively, where, *t* denotes time. Meanwhile, the three-dimensional vector function *r*(*t*) represents the line-of-sight between the optical center of the camera and each point in time. The three-dimensional vector function *u*(*t*) represents the unit vector of *r*(*t*), and scalar function *τ*(*t*) represents the length of *r*(*t*).

Based on this problem description, the camera parameters and the position of the point in the image, *p*(*t*) and *u*(*t*) are known, and the unknown *q*(*t*) needs to be solved. Obviously, if the polynomial of time with limited orders is used for description of *q*(*t*), then:

(1) $$q\left(t\right)=A_{q}{\left[t^{0},t^{1},\ldots ,t^{N}\right]}^{T}$$

where *A_q_* is a unknown quantity to be solved, which represents the 3(*N* + 1) coefficient matrix of polynomial with finite *N* orders used to describe *q*(*t*), *N* is the order of the polynomial. When *t* denotes time, the Equation (1) can express a different motion law of a point. For example, when *N* = 0, it means that the point is static; when *N* = 1 and *N* = 2, this indicates the point is in uniform motion and uniformly accelerating motion, respectively.

Assume *a_(i,j)_* is the element of row *i* and column *j* of the coefficient matrix *A_q_*, then the Equation (1) can also be written in another form:

(2) $$q(t)={\left[q_{x}(t),q_{y}(t),q_{z}(t)\right]}^{T}={\left[\sum_{ka=0}^{N}t_{}^{ka}a_{\left(1,ka+1\right)},\sum_{ka=0}^{N}t_{}^{ka}a_{\left(2,ka+1\right)},\sum_{ka=0}^{N}t_{}^{ka}a_{\left(3,ka+1\right)}\right]}^{T}$$

Based on the geometric relationship shown in Figure 1, it is obvious a basic equation exists as follows:

(3) q(t)=p(t)+τ(t)u(t)

Let:

(4) $$u(t)={\left[u_{x}(t),u_{y}(t),u_{z}(t)\right]}^{T}$$

and:

(5) $$p(t)={\left[p_{x}(t),p_{y}(t),p_{z}(t)\right]}^{T}$$

then:

(6) $$q(t)=\{\begin{array}{c} q_{x}(t)=\sum_{ka=0}^{N}t_{}^{ka}a_{\left(1,ka+1\right)}=p_{x}(t)+\tau (t)u_{x}(t)\\ q_{y}(t)=\sum_{ka=0}^{N}t_{}^{ka}a_{\left(2,ka+1\right)}=p_{y}(t)+\tau (t)u_{y}(t)\\ q_{z}(t)=\sum_{ka=0}^{N}t_{}^{ka}a_{\left(3,ka+1\right)}=p_{z}(t)+\tau (t)u_{z}(t) \end{array}$$

Equations (3) and (6) indicate the line-of-sight at time *t*, where the unknown quantity is the coefficient matrix *A_q_*, and each element characterizes the displacement, velocity, acceleration and other motion parameters of the point with a total number of 3(*N* + 1). Two independent equations could be listed for each time or each image. When the number of images is greater than 3(*N* + 1)/2, the equations may have a definite solution.

It is notable that the *N* value (order) of the polynomial is required before the equation can be solved, though the trajectory of a point’s motion is unknown. During the solution of the equation, the motion order could be approximately determined based on *a priori* knowledge of the laws of motion for actual objects. The biggest advantage in this description of motion is that the equations are linear with any given value of *N*, which facilitates the process of solving the equation.

**Figure 2:**
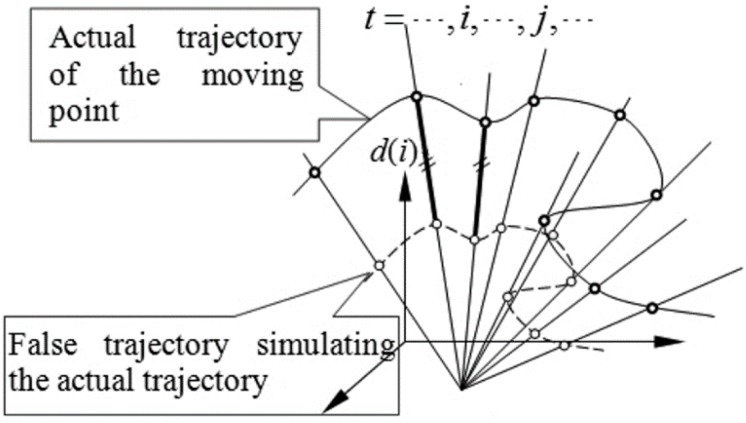
Failure to determine the actual trajectory when lines-of-sight intersect at one point.

However, the three-dimensional information of point motion cannot be reconstructed for all situations which have an image number greater than 3(*N* + 1)/2. Reference [13] list two situations in which a definite solution for point motion cannot be determined:

(1) When the moving point can be completely described by a three-dimensional vector function of time with finite order, and if the trajectory of the camera’s optical center can be completely described by a three-dimensional vector function with an order below that of the point, the definite solution can’t be determined.
(2) When all the lines of sight intersect at one point, the definite solution can’t be determined, as shown in Figure 2.

### 2.3. Required Conditions for Definite Solution of Point Motion

The existence of a unique solution is essential in the study of reconstruction of a moving point, while we have not solved the problem before. If a complete guideline for the existence of solutions cannot be found, the problem of a moving point reconstruction can’t be solved, especially for reconstruction of complex one.

In this paper, the two required conditions for the existence of definite solution for a moving point are proposed based on the relationship of the motion between the point and the camera. First, let ϖ[x(t)] denotes the order *N* of the polynomial *x*(*t*) and *N*_1_, *N*_2_ denotes the order of the polynomial *x_1_*(*t*), *x_2_*(*t*), respectively. Then, it is easy to calculate as follows:

(7) ϖ[−x(t)]=ϖ[x(t)]=N

and:

(8) $$\{\begin{array}{c} 0\leq \varpi [x_{1}(t)+x_{2}(t)]\leq N_{1},\text{if }N_{1}=N_{2}\\ \varpi [x_{1}(t)+x_{2}(t)]=\max(N_{1},N_{2}),\text{if }N_{1}\neq N_{2} \end{array}$$

and:

(9) $$\varpi [x_{1}(t)x_{2}(t)]=N_{1}+N_{2}$$

**Theorem 1.** *(line of sight conditions) Let N_q_, Nu denote the order of the three-dimensional vector function q(t) which approximately describes the moving point and the order of the unit vector u(t) of the line of sight, respectively. The basic Equation (3) or Equation (6) have a unique solution with Nq order if and only if N_u_ is greater than N_q_*.

Note: *N_u_* may be a finite number or infinite number, because the actual change in the direction of the line of sight cannot always be completely described by a polynomial with a finite order. As previously described, *N_q_* requires a preset finite value, and it decides that the number of unknowns is 3(*N_q_* + 1).

**Sufficient Proof.** For the sufficiency, its proof is: when *N_u_* is greater than *N_q_*, the basic Equation (3) or Equation (6) have a unique solution with *N_q_* order. Its reverse proposition is: when the basic Equation (3) or Equation (6) does not have a definite solution, *N_u_* is less than or equal to *N_q_*. The original proposition is proved if the reverse can also be proved. No definite solution exists in two situations: one is when there is no solution, and the other is when multiple solutions exist. Since the trajectory of the object is a natural solution of Equation (3), the basic Equation (3) or Equation (6) must also have a solution. Therefore, if we can prove that there is only one solution instead of multiple solutions, the establishment of the proposition can be proved. Suppose that *q*_1_(*t*) and *q*_2_(*t*), which are two different *Nq*-order three-dimensional vector functions with unknown factors, are the two *N_q_*-order solutions of the Equation (3), namely:

(10) $$\varpi [q_{1}(t)]=\varpi [q_{2}(t)]=N_{q}$$

where:

(11) $$q_{1}(t)-q_{2}(t)\neq 0$$

Substitute *q*_1_(*t*) and *q*_2_(*t*) into the Equation (3), eliminating *p*(*t*):

(12) $$q_{1}(t)-q_{2}(t)=[\tau_{1}(t)-\tau_{2}(t)]u(t)\neq 0$$

From the inequality Equation (8), we get:

(13) $$0\leq \varpi [q_{1}(t)-q_{2}(t)]\leq N_{q}$$

and:

(14) $$\varpi [[\tau_{1}(t)-\tau_{2}(t)]u(t)]=\varpi [\tau_{1}(t)-\tau_{2}(t)]+\varpi [u(t)]\geq N_{u}$$

Substituting Equation (14) into Equation (12), we get:

$$\varpi [[\tau_{1}(t)-\tau_{2}(t)]u(t)]=\varpi [\tau_{1}(t)-\tau_{2}(t)]+\varpi [u(t)]\geq N_{u}$$

(15) $$N_{u}\leq \varpi [[\tau_{1}(t)-\tau_{2}(t)]u(t)]=\varpi [q_{1}(t)-q_{2}(t)]\leq N_{q}$$

Namely:

(16) $$N_{u}\leq N_{q}$$

Then, the reverse proposition is affirmative, so the original proposition is affirmative, and the sufficiency is proved.

**Necessity Proof.** Its proof is: when the equation has a unique solution with an *Nq* order, *Nu* is greater than *Nq*. Let Nq-order three-dimensional vector function *q*(*t*) is the unique solution of Equation (3), then:

(17) $$\varpi [q(t)]=N_{q}\geq 0$$

Let three-dimensional vector function *q*'(*t*) denote an arbitrary curve, which meets the constraints of line of sight and does not coincide with *q*(*t*). Based on the condition of the necessity, if *q*'(*t*) is not an *Nq*-order solution of the basic equation, then its order must meet the following requirements:

(18) $$\varpi [q'(t)]=N_{q'}>N_{q}$$

Otherwise, *q*'(*t*) must satisfy the basic function, namely, be the solution. For the same time *t*, *q*'(*t*) and *q*(*t*) denote two points on the same line of sight, then:

(19) q'(t)=q(t)+τ'(t)u(t)

where, *τ*'(*t*) is a one-dimensional scalar function. The order of the function can be expressed as:

(20) $$\varpi [q'(t)]=N_{q'}=\varpi [q(t)+\tau '(t)u(t)]$$

Let the order of *τ*'(*t*) is *N_τ_*_'_, based on Equations (7)–(9), we get:

(21) $$\{\begin{array}{c} 0\leq N_{q'}\leq N_{q},\text{if }N_{u}+N_{\tau '}=N_{q}\\ N_{q'}=\max(N_{q},N_{u}+N_{\tau '}),\text{if }N_{u}+N_{\tau '}\neq N_{q} \end{array}$$

Substitute Equation (21) into Equation (18), we can further get:

(22) $$N_{q'}=N_{u}+N_{\tau '}>N_{q}$$

Because *N_τ_*_'_ ≥ 0 and the Equation (22) must be established for any value of *N_τ_*_'_, there must be:

(23) $$N_{u}>N_{q}$$

The necessity is proved.

**Deduction 1.** *(Camera motion conditions) Let N_q_, N_p_ denote the order of the three-dimensional vector function q(t) which describes the moving point, and the order of the three-dimensional vector function p(t) which describes the trajectory of the camera’s optical center, respectively, while the order of the scalar function τ(t) which describes the changes of the norm of the line of sight vector r(t) is N_τ_, if and only if N_p_ is greater than the sum of N_q_ and N_τ_, the basic Equation (3) or Equation (6) has a unique solution with N_q_ order.*

Note: *N_p_* and *N_τ_* may be a finite number or infinite number, because the actual trajectory of camera’s optical center and the change of the norm of line of sight vector are not always be completely described by a polynomial with a finite order. However, *N_q_* is a preset finite value, and the number of unknown is 3(*N_q_* + 1).

**Necessity Proof.** For the necessity, its proof is: when the Equation (3) has a unique solution with *N_q_* order, *N_p_* > *N_p_* + *N_τ_*. By Theorem 1, we get:

(24) $$N_{u}>N_{q}$$

Based on Equation (3), we get:

(25) $$\varpi [p(t)]=N_{p}=\varpi [q(t)+\tau (t)u(t)]$$

Based on Equation (24), we get:

(26) $$N_{u}+N_{\tau }>N_{q}$$

Based on the order nature, we get:

(27) $$N_{p}=\max(N_{q},N_{\tau }+N_{u})=N_{\tau }+N_{u}>N_{\tau }+N_{q}$$

The necessity is proved.

**Sufficient Proof.** For the sufficient, its proof is: when *N_p_* is greater than the sum of *N_q_* and *N_τ_*, the basic Equation (3) or Equation (6) has a unique solution with *N_q_* order. Based on the Equation (25) and the order nature, we get:

(28) $$\{\begin{array}{c} 0\leq N_{p}\leq N_{q},\text{if }N_{u}+N_{\tau }=N_{q}\\ N_{p}=\max(N_{q},N_{u}+N_{\tau }),\text{if }N_{u}+N_{\tau }\neq N_{q} \end{array}$$

Based on the sufficient conditions, we get:

(29) $$N_{p}>N_{\tau }+N_{q}$$

Then:

(30) $$N_{p}=\max(N_{q},N_{\tau }+N_{u})>N_{\tau }+N_{q}$$

Based on the Equation (30), it can be deduced that:

(31) $$N_{\tau }+N_{u}>N_{\tau }+N_{q}\Rightarrow N_{u}>N_{q}$$

Based on Theorem 1, it has a unique solution with *N_q_* order, thus the sufficiency is proved.

When a higher order polynomial, compared with the one of camera, denotes the motion of a moving point, if it does not meet the conditions described above, the trajectory of the moving point cannot be determined. Naturally, a model with a lower order is considered to describe the moving point. Although actual physical situations, such as the motion of the camera’s optical center, changes of line of sight, are the same as before, that is, their functional models of characterization are unchanged, the required condition is likely to be met again because the model of the moving point is simplified, and the number of unknowns in the listed equations is reduced correspondingly. Thus, when the initial functional model for a moving point is complex and definite solutions cannot be determined, results may be obtained by simplifying the model. The experiment described below shows the reasonability of simplification which can still guarantee the accuracy of the model.

### 2.4. Solving Method for Position and Orientation of Rigid Body in Motion

The above solves the problem that how to determine the trajectory and motion parameters of a point with a monocular moving camera. Next, we naturally think of whether or not the orientation of the moving rigid body can also be determined through the monocular moving camera. If so, how to determine it?

Generally, the orientation of a rigid body can be determined by “PnP” methods if more than three known non-collinear points on it. Many algorithms, such as an orthogonal iteration algorithm [14], work quite well. If the homonymous points in different images are available but unknown, the problem can be solved by the methods of “Multiple view geometry” [12]. This section will discuss two insoluble problems by the above:

(1) How to determine the orientation partially or totally with less than three known points on rigid body?
(2) How to determine the orientation with less than eight unknown points on rigid body?

Solution of orientations can be easily derived from the proposed method for trajectory of the point. If the trajectories of all feature points on a rigid body have been solved, the orientations at any moment can be worked out by the positons of points at the same time. Furthermore, one additional constraint that the distances among feature points on a body are constant is helpful to increase stability and to optimize the result of orientations. The process of calculation for every point on the body is independent. The estimated distances between points would not be fixed. The optimization with the condition of distances invariability can be done by Levenberg-Marquard algorithm.

The main procedure includes two steps:

(1) Solve the initial trajectories value of the points.
  Assuming the number of points on the moving rigid body is *M*, the three-dimensional vector *q_i_*(*t*) denote the position of the *i*-th point at time *t*. Each initial value ${\hat{q}}_{i}(t)$ of *q_i_*(*t*) can be solved by Equation (3). Technically, the orientations can be estimated now.
(2) Optimize the results with distances invariability to update the value of the trajectory.
  Based on the nature of rigid body motion, the distance between each point on the rigid body remains unchanged, namely:
  (32) $$||q_{i}(t)-q_{j}(t)||=d_{ij}$$
  where, 1< *i*< *M*, 1< *j*< *M*, *I* ≠ *j*. *d_ij_* is the distance between the *i*-th point and the *j*-th point, which is a constant and does not change over time. These constants are taken as constraints to improve the solution accuracy of the position and orientation. The two cases are discussed below, respectively:
  (A) Known relationship between points
    In this situation, *d_ij_* is known, and Equation (32) is taken as a conditional equation. The correct equation can be established based on the initial value ${\hat{q}}_{i}(t)$ and the known observations, for the solution of *q_i_*(*t*) by Levenberg-Marquard method.
  (B) Unknown relationship between points
    For this case, *d_ij_* is a constant but unknown. The adjustment process is as follows:
    - The initial values ${\hat{d}}_{ij}(t)$ of *d_ij_* at different time are calculated by ${\hat{q}}_{i}(t)$, then the mean value *md_ij_* and standard deviation *sd_ij_* of ${\hat{d}}_{ij}(t)$ over the time can be obtained. The values of *d_ij_* are set to the mean value of ${\hat{d}}_{ij}(t)$, *i.e.*, *md_ij_*.
    - Now, *d_ij_* is as known, Refer to case (A), the optimization of orientation can be obtained.

## 3. Experiments

The experiment are conducted in four parts: (1) reconstruction of 2D trajectory; (2) reconstruction by segmentation or by reduced order; (3) reconstruction of 3D trajectory; (4) reconstruction of orientations.

### 3.1. Reconstruction of 2D Trajectory

**Figure 3:**
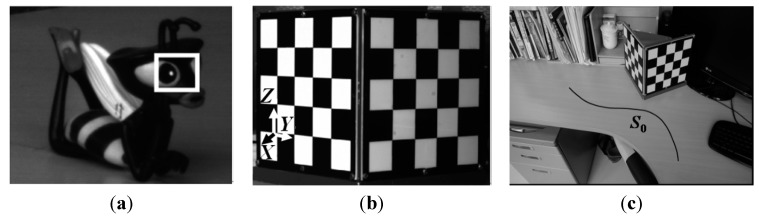
Experimental scene of 2D trajectory. (**a**) An object point; (**b**) A chessboard for the camera calibration and the definition of coordinate system; (**c**) The guiding line S_0_.

In this experiment, the object is a toy bee, and the feature point is the center of the white spot in the left eye on the object, as shown in Figure 3a. The true distance between the point and the table plane is 26.5 mm, which is measured by a ruler with an accuracy of 1.0 mm. Camera parameters are calibrated through the three-dimensional chessboard as shown in Figure 3b, as well as the definition of coordinate system. The doll moves along the curve S_0_, as shown in Figure 3c, and 12 images are captured during the motion process.

**Figure 4:**
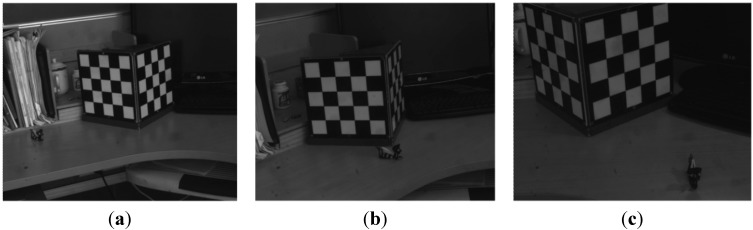
The part images of sequence for 2D reconstruction. (**a**) The 1st image; (**b**) The 5th image; (**c**) The 11thimage.

**Figure 5:**
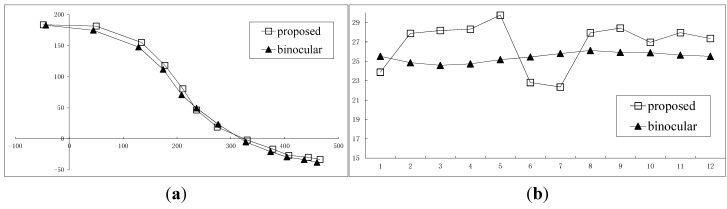
The results of 2D reconstruction. (**a**) XOY plane; (**b**) Z direction over the time.

Several images of the sequence are shown in Figure 4. The overall distance travellrd by the doll is about 540 mm, and the distance from the camera to the doll is around 1100 mm. In order to illustrate the accuracy, the trajectory of the guiding line S_0_ rebuilt by the traditional binocular measurement is used to compare with the result obtained by the proposed method. It shows that the results of guiding line S_0_ are consistent with the trajectory of the feature point on the toy bee in the XY direction, as shown in Figure 5a, and in the Z direction, as shown in Figure 5b. With further analysis, the mean value of the Z direction by binocular reconstruction is 25.4 mm, while the same value by proposed is 26.8 mm, for a difference between them of 1.4 mm. The mean and the standard deviation of the space residuals relative to the object distance are only 0.6% and 3.0%, respectively.

### 3.2. Reconstruction by Segmentation or by Reduced Order

**Figure 6:**
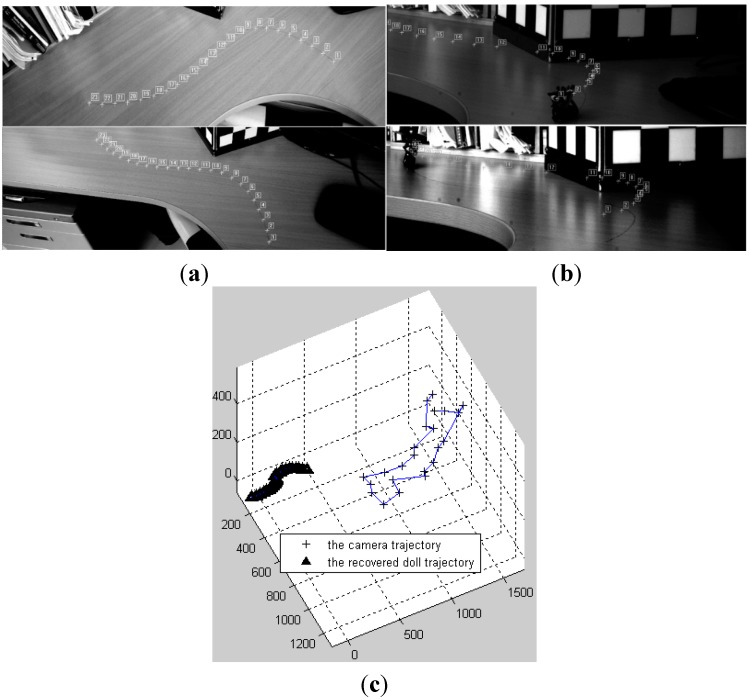

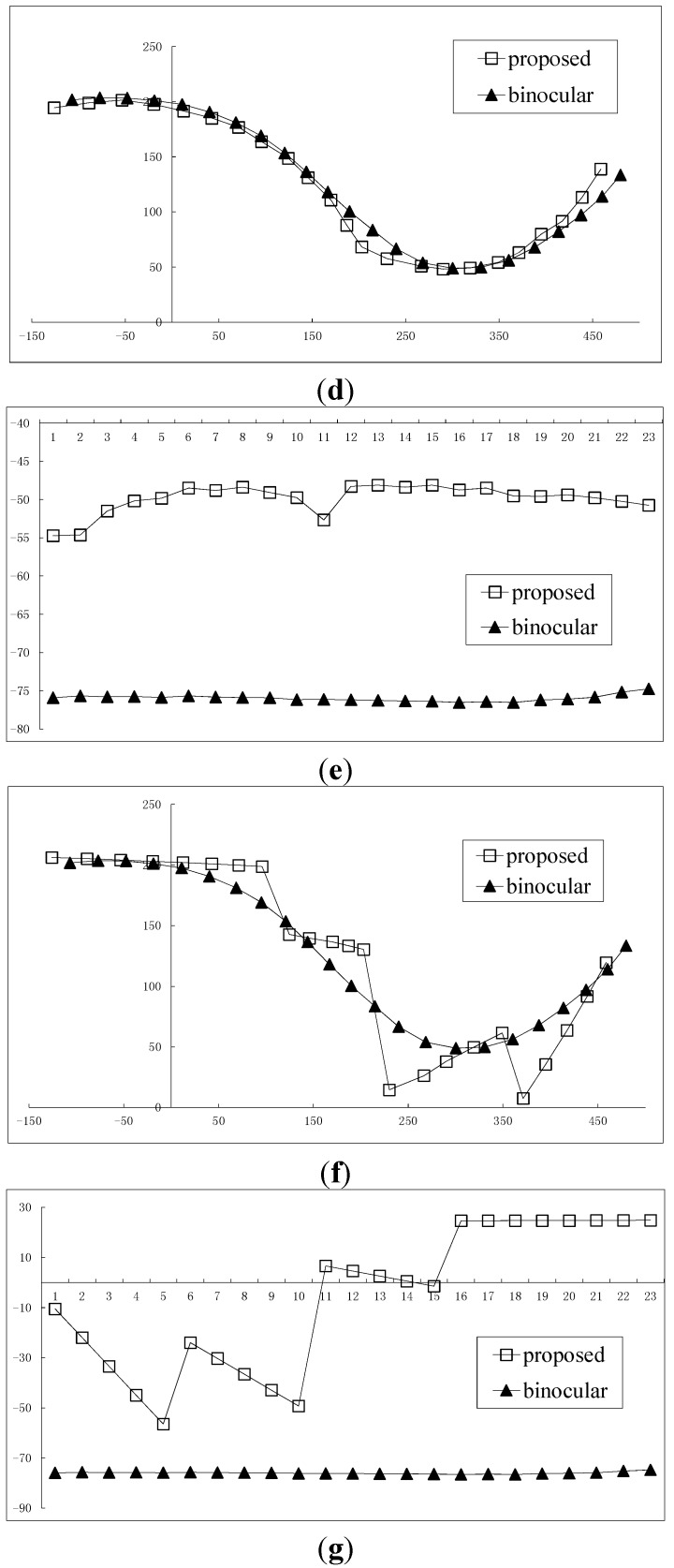
Reconstruction by segmentation. (**a**) The guiding line images taken by the two cameras; (**b**) The reprojection by the results of proposed method on the first image (above) and the last image (below); (**c**) The camera trajectory and the rebulit one of the point; (**d**) and (**e**) The proposed and binocular results in the XOY plane and the Z direction, respectively; (**f**) and (**g**) The proposed with four line section approximation and binocular results in the XOY plane and the Z direction, respectively.

Figure 6a shows the guiding line images taken in two directions by binocular cameras. Figure 6b is the reprojection of the monocular reconstruction result on the first image (above) and the last image (below). Figure 6c is a diagram showing the space relationship of the camera movement and the monocular reconstruction results. Figure 6d,e shows the comparisons of monocular and binocular results in the XOY plane and the Z direction, respectively. The results obtained from the two methods agree quite well. Figure 6f,g shows the reconstruction results taking straight lines as the approximation of the object trajectory in four sections. It shows the results obtained from linear reconstruction are a little bit poorer.

Reconstruction by segmentation has a great significance. In general, due to unsatisfactory olvable conditions, the algorithm will be invalid at some situations, such as where part of the curves self-intersect. For example, a circle is a kind of self-intersection curve. Circles can be expressed by trigonometric functions which can be represented by a polynomial of infinite order. The solvable conditions cannot be satisfied by any camera motions. An ellipse is the same as a circle. Under this circumstance, the solvable condition can be satisfied by segmenting the circle into two parabolas, approximately. The reconstruction procedure for the two parabolas is similar to the segmentation above.

**Figure 7:**
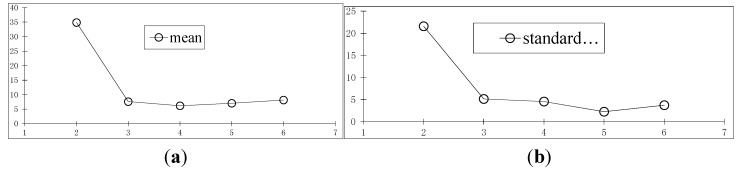
The rebuilding errors of the polynomials with the different orders. (**a**) The curve for mean values of residuals; (**b**) The standard deviation curve of residuals.

Reconstruction by reduced order is also important. Figure 6f,g shows that the result is poor when first-order linear motion is used to describe the high-order motion. Next, the time vector functions with an order from two to six are used to describe the trajectory of the object and reconstruct the point, respectively. The results are also compared with the results of binocular reconstruction, as shown in Figure 7. The horizontal axis is the order value, and the vertical axis is the residual compared with the results of the binocular reconstruction. Figure 7a is the curve for mean values of residuals, and Figure 7b is the standard deviation curve of residuals. The unit of residuals is mm.

When vector functions with different orders are used to describe the movement of objects, most of the reconstruction results retain a certain amount of precision, and when the description is clearly not able to characterize the actual movement law, the error is significantly larger. For example, a straight line does not fit this situation but the results of other order are quite good. It shows that, when the solving conditions cannot be met with the high-order vector functions, the vector function with appropriate lower order can still ensure a certain degree of accuracy.

### 3.3. 3D Trajectory Reconstruction

**Figure 8:**
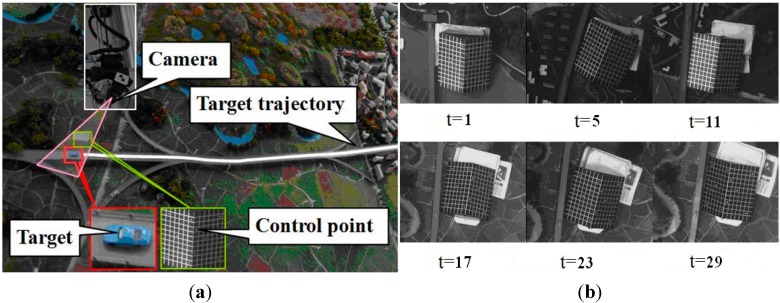
Experimental scene of 3D trajectory. (**a**) An apparatus for the reconstruction of toy car trajectory; (**b**) The part images of sequence for 3D reconstruction.

In this experiment, the target object is a toy car, and the feature point is the center of the windshield in front of the car. Camera orientations at every moments are estimated by the white control points on a chessboard, which coordinate values at every moment are obtained by a total station with an accuracy of about 2 mm. Camera positions are located at the tip of an opposite vertical angle and are also get by the total station. The car moves along the viaduct (white line) in a sand table. These are all shown in Figure 8.

**Figure 9:**
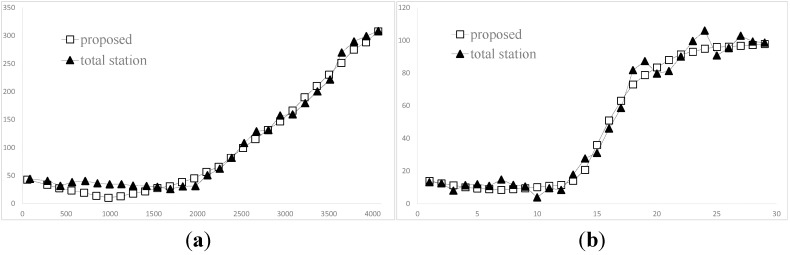
The results of 3D reconstruction. (**a**) XOY plane; (**b**) Elevation over the time.

**Figure 10:**
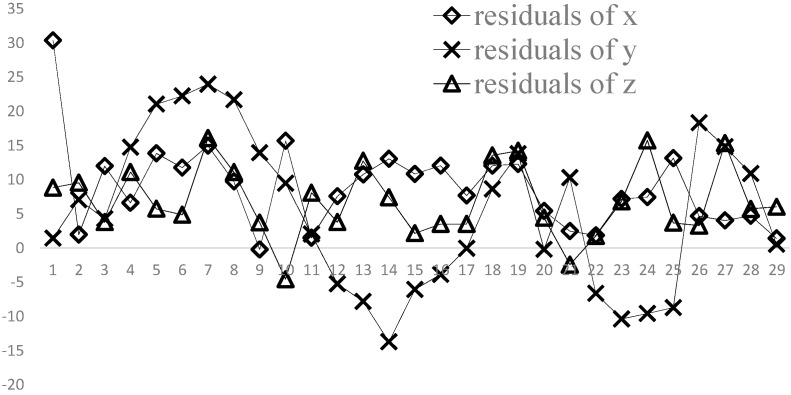
The residuals of 3D reconstruction at every moment.

The overall travel distance in the horizontal plane is about 4000 mm, and the biggest change in elevation is about 100 mm. The distance between the camera and the car is about 2000 mm. During the process, 29 images are captured, and the positions of the point are also located by the total station, meanwhile. Figure 9 shows the comparision of two kinds of results. The residuals between the total station and the proposed in the three directions are shown in Figure 10. The standard deviations are 4.7 mm, 11.6 mm, 5.6 mm, respectively.

### 3.4. Reconstruction of Orientations

#### 3.4.1. Two Points Reconstruction Experiment with Known Distance

**Figure 11:**
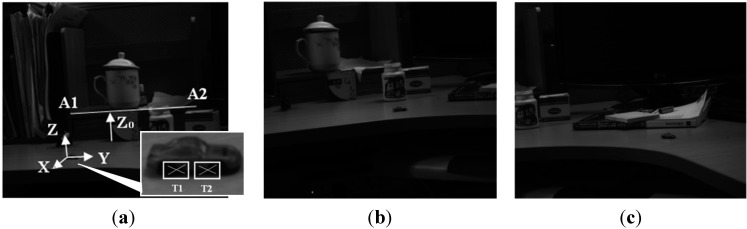
The image sequence of the moving car. (**a**) The 1st image and the coordinate determination; (**b**) The 5 th image; (**c**) The 11th image.

Eleven images were captured in this experiment. Figure 11 shows some of them. The camera parameters of each image are obtained from the “static scene”, and a scale is given as shown in Figure 11a. The distance between A1 and A2 was measured with a ruler at 300 mm. As Figure 11a, the coordinate is determined by the right hand rule as follows: the point T1 at the car in the first image is taken as the original point, the direction of T1T2 is set as Y-axis, and the direction Z_0_ of the ink cartridge in the image is set as the Z direction.

**Figure 12:**
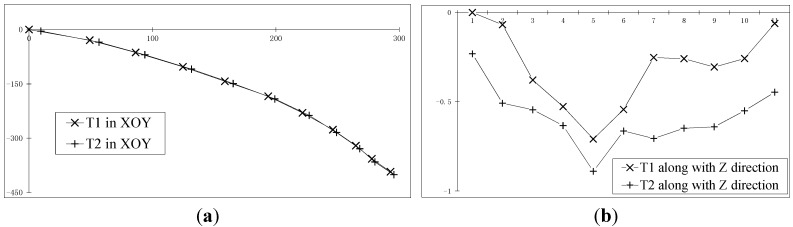

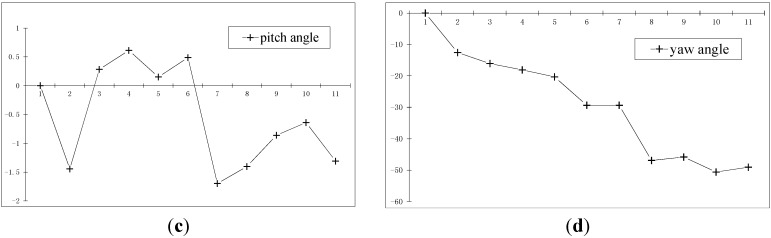
The recovery of the moving car. (**a**) The trajectory reconstruction results of two moving points on the car in the XY plane; (**b**) Those in the Z direction; (**c**) The measurement results of the pitch angle of the car; (**d**) The measurement results of the yaw angle of the car.

The measurement results could be obtained through the method discussed in Section 2.4. Figure 12a,b is the trajectory reconstruction results of two moving points on the car in the XY plane (the horizontal axis is Y, the vertical axis is X) and in the Z direction (the vertical axis is Z, the horizontal axis is the image number), respectively. Figure 12c,d is the measurement results of the pitch angle and yaw angle of the car, respectively.

#### 3.4.2. Two Points Reconstruction Experiment for Unknown Distance

**Figure 13:**
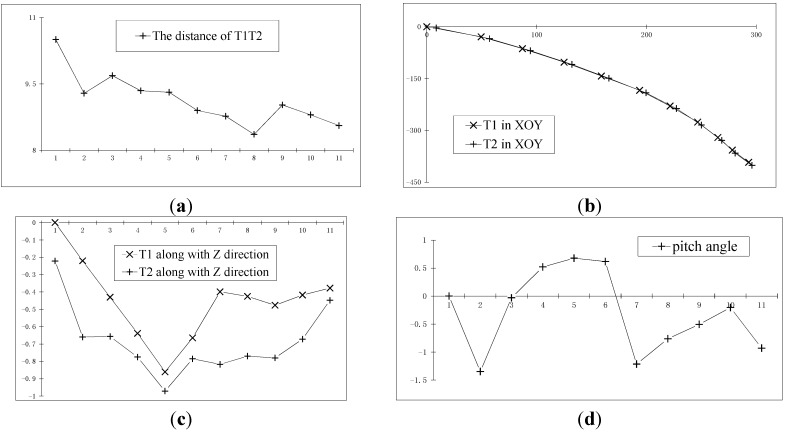

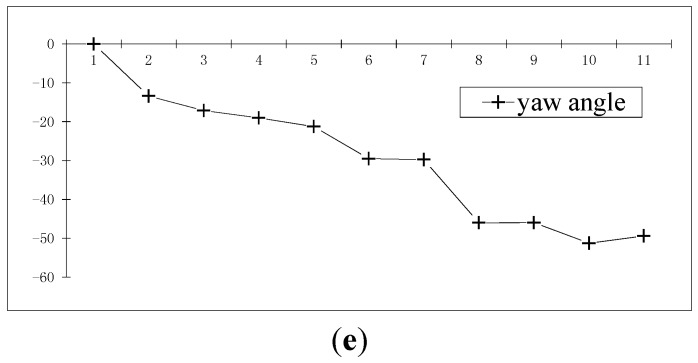
The recovery of the moving car. (**a**) The distance value of T1T2 at each point in time; (**b**) The trajectory reconstruction results of two moving points on the car in the XY plane; (**c**) Those in the Z direction; (**d**) The measurement results of the pitch angle of the car; (**e**) The measurement results of the yaw angle of the car.

The experimental images of Section 3.4.1 are still used in this section. It’s assumed that the distance between T1 and T2 is invariable but unknown. Based on the steps in Section 2.4., the trajectory of T1 and T2 are calculated, and the bundle adjustment method is applied. The calculated mean distance between T1 and T2 is 9.2 mm, with a standard deviation of 0.6 mm. Figure 13a shows the distance value of T1T2 at each point in time. Keeping the distance value of T1T2 unchanged and equal to 9.2 mm for the constraints, adjustment is applied. The calculation results are shown in Figure 13b–e, which are the trajectory reconstruction results of two moving points on the car in XY plane (the horizontal axis is Y, the vertical axis is X) and in the Z direction (the vertical axis is Z, the horizontal axis is the image number), and the measurement results of the pitch angle and yaw angle of the car, respectively.

In this section, the calculated mean distance is used to replace the actual measurement value for solving. The results are basically consistent with the results of Section 3.4.1 For example, the calculated variation of the overall yaw angle in Section 3.4.1 is 49.05°, while the corresponding value in this section is 49.38°, and the other corresponding quantities are basically the same.

#### 3.4.3. Multi-Points Reconstruction Experiment of Unknown Point Distances

**Figure 14:**
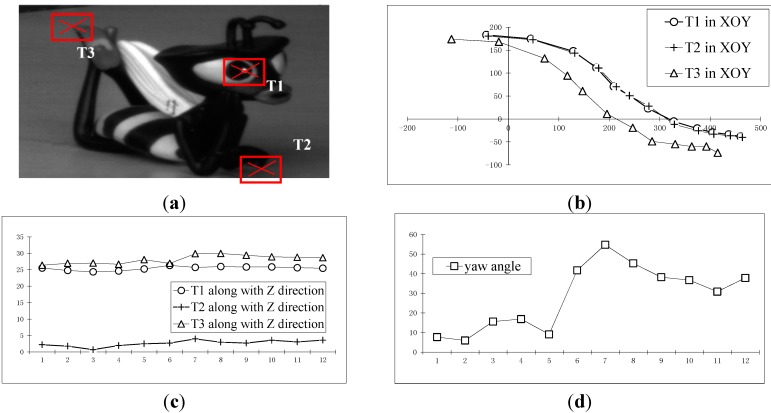

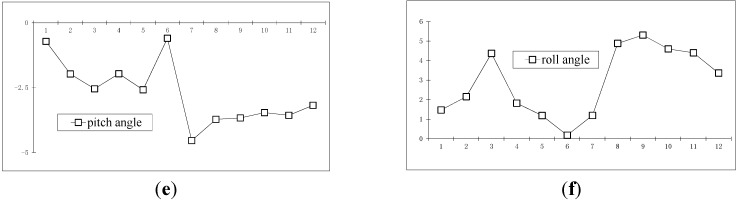
The recovery of the doll. (**a**) The points to be measured on the doll; (**b**) The trajectory reconstruction results of three moving points on the doll in the XY plane; (**c**) Those in the Z direction; (**d**) The measurement results of the pitch angle of the doll; (**e**) The measurement results of the yaw angle of the doll; (**f**) The measurement results of the roll angle of the doll.

The experimental images in Section 3.1 are used in this section, and two points to be measured are added based on the experiment in Section 3.1; see T1, T2, T3, as shown in Figure 14a. Figure 14b–f shows the results, which are the trajectory reconstruction results of three moving points on the doll in the XY plane (the horizontal axis is Y, the vertical axis is X) and in Z direction (the vertical axis is Z, and the horizontal axis is the image number), and the measurement results of pitch angle, yaw angle and roll angle, respectively.

## 4. Conclusions

In this paper, a three-dimensional reconstruction method for complex moving objects using a monocular moving camera is proposed. A complete proof for the existence and uniqueness of the solution is provided. The method assumes the object as a point or a rigid body for acquisition the trajectory and orientation, respectively. A finite order polynomial of time is used for approximation description of the movement law of the object. the object trajectory and speed, acceleration and other motion parameters as well as the orientation can be solved by linear equations, which resolves the problems that a monocular moving camera cannot reconstruct the trajectory of the complex moving point or calculate the orientation of the object. It greatly expands the applications of monocular moving camera on three-dimensional reconstruction for moving objects. Compared with other methods, the important features are:

Firstly, it greatly expands the range of applications. The trajectory triangulation method of Avidan and Shashua is limited to the point moving along straight lines or conics; the methods of Han and Kanade as well as Xu and others is limited to the trajectory of the points moving with uniform motion; the proposed method can reconstruct the trajectory, velocity, acceleration and other motion parameters as well as the three-dimensional orientation for almost any complex moving object.

Secondly, the solvable conditions are of more practical significance. The trajectory triangulation method requires at least five or nine images, and it requires that “the trajectory of the optical center of the camera and the trajectory of the object is not in the same quadratic surface”. Other methods do not discuss solvable conditions. In this paper, it requires the order of the vector functions of time which characterizes the vector of line of sight to be greater than the order of the similar functions for the moving objects. In an unprecise expression, it ask camera motion should be more complicated than the object. It is more practical in the path planning of camera motion for measurement.

Thirdly, the solution method is more concise. In the trajectory triangulation method, the problem in three-dimensional cartesian coordinate space is transformed into a problem in six-dimensional Plücker coordinate space for rebuilding the moving objects along a straight line. An initial value of the curve plane is required for an iterative solution when the object motion is along a conic. Other methods by the extended Kalman filtering need a good initial value or iteration. The proposed uses linear equations to solve motion parameters without any initial value, directly. For the orientation of the body target, multi points do not increase the complexity but provide a redundant constraint, and improve the accuracy.

Fourthly, when the solvable conditions are not met, the reduced-order method can be used to obtain an approximate solution and still maintain a quite good precision. It can not be solved by other methods.

The method can be applied to many areas such as target observation of UAVs, robot soccer systems, driverless cars, which tend to use additional equipment like laser rangefinders, ranging radars, *etc.*, and before were difficult to handle with only a monocular camera. Further studies will be mainly carried out in several directions:

(1) How to determine the optimal order of the object motion adaptively?
(2) How to find the optimal time period to calculate as a whole trajectory?
(3) How to get the optimal solution?
